# Disease severity predicts higher healthcare costs among hospitalized nonalcoholic fatty liver disease/nonalcoholic steatohepatitis (NAFLD/NASH) patients in Spain

**DOI:** 10.1097/MD.0000000000023506

**Published:** 2020-12-11

**Authors:** Manuel Romero-Gomez, Nandita Kachru, Meritxell Ascanio Zamorano, Josep Darba, Sanatan Shreay

**Affiliations:** a Hospital Universitario Virgen del Rocío; b SeLiver Group, Instituto de Biomedicina de Sevilla, Sevilla; c CIBERehd, Madrid; d University of Seville, Sevilla, Spain; e Gilead Sciences, Inc., Health Economics Outcomes Research, Foster City, USA; f BCN Health Economics & Outcomes Research; g Department of Economics, University of Barcelona, Barcelona, Spain.

**Keywords:** advanced liver diseases, comorbidities, costs, length of stay, NAFLD, NASH

## Abstract

The rising prevalence of nonalcoholic fatty liver disease (NAFLD) and nonalcoholic steatohepatitis (NASH) presents many public health challenges, including a substantial impact on healthcare resource utilization and costs. There are important regional differences in the burden of NAFLD/NASH, and Spain-specific data are lacking. This retrospective, observational study examined the impact of liver disease severity, comorbidities, and demographics on healthcare resource utilization and costs in Spain.

NAFLD/NASH patients in the Spanish National Health System's Hospital Discharge Records Database (1/1/2006 to 4/30/2017) were categorized into disease severity cohorts as NAFLD/NASH overall, NAFLD/NASH non-progressors, compensated cirrhosis (CC), decompensated cirrhosis (DCC), liver transplant (LT), or hepatocellular carcinoma (HCC). Patients were followed from index date until the earliest of 6 months, disease progression, end of coverage, death, or end of study. Within each cohort, pre- and post-index healthcare resource utilization and costs per patient per month (PPPM) were calculated.

A total of 8,205 patients (mean age 58.4; 54% male) were identified; 5,984 (72.9%) were non-progressors, 139 (1.7%) progressed to CC, 2,028 (24.7%) to DCC, 115 (1.4%) to LT, and 61 (0.7%) to HCC. Pre-index comorbidity burden was high across disease cohorts, and the frequency of comorbidities increased with disease severity. From pre- to post-index, average length of stay (LOS) increased significantly (23%–41%) as did all-cause PPPM costs (44%–46%), with significantly longer LOS and costs in patients with increasing disease severity.

Progression of NAFLD/NASH was associated with significantly higher costs and longer LOS. A coordinated approach is needed to manage resources and costs in Spain.

## 1 Introduction

Nonalcoholic fatty liver disease (NAFLD) is a leading cause of chronic liver disease worldwide,^[1–5]^ with disease severity ranging from simple steatosis to the combination of steatosis, inflammation, and hepatocyte ballooning that is characteristic of nonalcoholic steatohepatitis (NASH).^[6,7]^ NAFLD/NASH is widely recognized as the hepatic manifestation of metabolic syndrome,^[6,8,9]^ and affected patients are at increased risk of insulin resistance and type 2 diabetes mellitus,^[7,10–13]^ hyperlipidemia,^[7,12–14]^ obesity,^[13,15,16]^ and hypertension.^[7,12,13,15,17]^ With the progression of NAFLD to NASH, patients are also at substantially greater risk of liver fibrosis and advanced liver diseases including compensated cirrhosis (CC) and decompensated cirrhosis (DCC);^[7,13,15,18]^ hepatocellular carcinoma (HCC);^[4,7,13,15,18,19]^ liver transplant (LT);^[20,21]^ and death.^[7,13,22,23]^

The prevalence of NAFLD/NASH is projected to increase worldwide as obesity and diabetes rates continue to rise and the population of the world ages, with current estimates suggesting that 25% of the global adult population has NAFLD, and 3% to 5% are affected by NASH.^[3,24]^ A dynamic Markov model, based on data from national reports and surveillance activities combined with a literature review and consultation with experts, characterized trends in NAFLD prevalence and progression in 8 countries including China, France, Germany, Italy, Japan, Spain, the United Kingdom (UK), and the United States (US). By 2030, the NAFLD population is projected to increase by 18.3% to 100.9 million cases worldwide, with a 15% to 56% increase in the worldwide prevalence of NASH. Within Europe, the highest prevalence of NAFLD (including NAFL and NASH) in 2030 is predicted for Italy at 29.5% followed by 27.6% for Spain, while the highest prevalence of NASH is estimated for Italy (6.3%), Germany (6.0%), and Spain (5.9%) versus 5.0% in France.^[1]^

As the prevalence of NAFLD and NASH rises, the associated health, economic, and personal impacts on patients, family members, society, and healthcare delivery systems are expected to increase substantially.^[5,25–28]^ NAFLD/NASH has a significant and negative effect on patients’ quality of life and wellbeing.^[28–32]^ Affected patients experience higher rates of depression than patients with hepatitis B and the general population,^[33]^ and are at increased lifetime risk of major depressive disorder.^[34]^

NAFLD/NASH also imposes a substantial burden on healthcare resource utilization and costs.^[5,26,27,35,36]^ Annual direct medical costs for all incident and prevalent cases of NAFLD were estimated at €35 billion in 2016 for Germany, France, Italy, and the UK, with societal costs ranging from €31 billion in the UK to €75.7 billion in France.^[27]^ Healthcare resource utilization and costs are significantly higher for patients who progress to more severe liver disease compared with NAFLD/NASH and no progression.^[25,37,38]^ The impact of NAFLD/NASH on healthcare systems and direct costs is projected to increase^[5,36]^ as the number of affected persons rises in Europe and worldwide.

Accurate estimates of the prevalence and burden of NAFLD/NASH are required for the design, implementation, and evaluation of a strategic public health response to address the growing impact of NAFLD/NASH in specific countries and regions of the world.^[5,39,40]^ Real-world, population-based data can accurately characterize the prevalence, progression, and economic impact of NAFLD and NASH within specific countries and regions, which, in turn, can inform health policy.^[41]^ To the authors’ knowledge, there are no existing population-based studies that provide real-world estimates of the current impact of NAFLD/NASH in Spain. Thus, the objectives of this study were to examine the comorbidity burden, healthcare resource utilization, and associated costs among NALFD/NASH patients with advanced liver diseases in Spain.

## 2 Methods

### 2.1 Design and data source

A longitudinal, retrospective cohort study using data from the Spanish National Health System′s Hospital Discharge Records Database (Conjunto Mínimo Básico de Datos or CMBD) for the period 01 January 2006 through 30 April 2017 was performed. Under the supervision of the Spanish Ministry of Health, the CMBD compiles information from 192 private and 313 public hospitals in all regions of Spain. The database contains patient-level information on demographic characteristics, primary and secondary diagnoses, readmissions, healthcare costs, dates of admission and discharge, and mean length of stay (LOS) for hospitalized patients. Information for this study was available for more than 36 million patients between 2006 and 2017.

Patient-level data were anonymized in accordance with the principles of Good Clinical Practice and the Declaration of Helsinki. According to Spanish data protection regulations, patient consent and approval by an institutional review board or ethics committee were not required for de-identified patient information.

### 2.2 Sample selection

Patients eligible for inclusion in this analysis were ≥18 years with at least 1 inpatient claim between January 1, 2006 and April 30, 2017 for a known diagnosis of NAFLD or NASH. A diagnosis of NAFLD or NASH was based on the International Classification of Diseases, Ninth Revision, Clinical Modification (ICD-9-CM) codes 571.8 and 571.9 and the International Classification of Diseases, Tenth Revision, Clinical Modification (ICD-10-CM) codes K76.0, K75.81. ICD-10-CM codes were first implemented in Spain in January 2016, and this study included a time period that preceded and followed the implementation of ICD-10-CM codes. Therefore, both ICD-9-CM and ICD-10-CM diagnosis codes for NAFLD and NASH were utilized.

Patients were excluded if, at any time during the study period, they were diagnosed with other etiologies of liver diseases (including viral hepatitis [hepatitis A, B, C, D, E], toxic liver disease, autoimmune hepatitis, Wilson's disease, Gaucher disease, lysosomal acid lipase deficiency, alcoholism including alcoholic liver disease, primary biliary/sclerosing cholangitis, or hemochromatosis) or human immunodeficiency virus. The list of ICD-9-CM and ICD-10-CM codes for identification of patients with NAFLD/NASH, CC, DCC, LT, and HCC are contained in Appendix 1.

### 2.3 Study cohorts

The date of the first claim associated with the diagnosis of NAFLD/NASH was defined as the index date. Eligible patients were required to have continuous medical and prescription coverage for at least 6 months before and at least 1 month following the NAFLD/NASH index date. The presence of ICD-9-CM or ICD-10-CM codes for advanced liver diseases following the NAFLD/NASH index date resulted in the categorization of patients into 1 of 6 non-mutually exclusive cohorts based on the severity of liver disease. The cohorts were NAFLD/NASH overall, NAFLD/NASH non-progressors, CC, DCC, LT, and HCC.

The baseline or pre-index period was defined as the 6-month interval immediately preceding the index date. The follow-up or post-index period for eligible patients was defined as the time from the index date to one of the following, whichever was earliest:

1) 6 months after the index date,
2) progression to a different disease severity cohort,
3) end of coverage,
4) death, or
5) end of the study period.

### 2.4 Endpoints

The primary endpoints were all-cause healthcare resource utilization and costs for the pre-index and post-index periods for each of the liver disease severity cohorts. Healthcare costs were expressed as per patient per month (PPPM) values and included outpatient, inpatient, and pharmacy expenditures. Costs were adjusted to 2017 euro. Measures of healthcare resource utilization included the number of readmissions per patient and average LOS per admission.

Patient age, sex, and region of residence as well as comorbid health conditions were characterized for the pre-index period for each of the disease severity cohorts. ICD-9-CM and ICD-10-CM codes were used to identify comorbidities, which included abdominal pain, anemia, bariatric surgery, cardiovascular disease (CVD), diabetes mellitus (type 1 and type 2), dyspepsia, hyperlipidemia, hypertension, insomnia, obesity, renal impairment, sleep apnea, thyroid disease, and vitamin D deficiency.

### 2.5 Statistical analysis

Descriptive statistics were calculated for all variables, including frequencies and percent responses for categorical variables and mean, median, and standard deviation for continuous variables. Healthcare resource utilization and costs were analyzed as continuous variables.

Chi-squared tests were used for the analysis of differences between categorical variables, and paired *t* tests were used for comparisons of pre- and post-index healthcare resource utilization and costs. *P* values <.05 were considered significant. Generalized linear models (GLM) with gamma error distribution and log-link function evaluated the incremental cost burden after adjustment for severity of liver disease and pre-index demographic and clinical characteristics. An important advantage of the GLM approach is that the log-transformed model is not subject to the assumptions that are necessary for a least-squares regression model, which are usually not held with cost data. Explanatory variables included severity of liver disease, age, sex, geographic region, and comorbid conditions. All statistical analysis was performed with SAS version 9.4 [SAS Institute, Cary, NC].

## 3 Results

**Figure 1:**
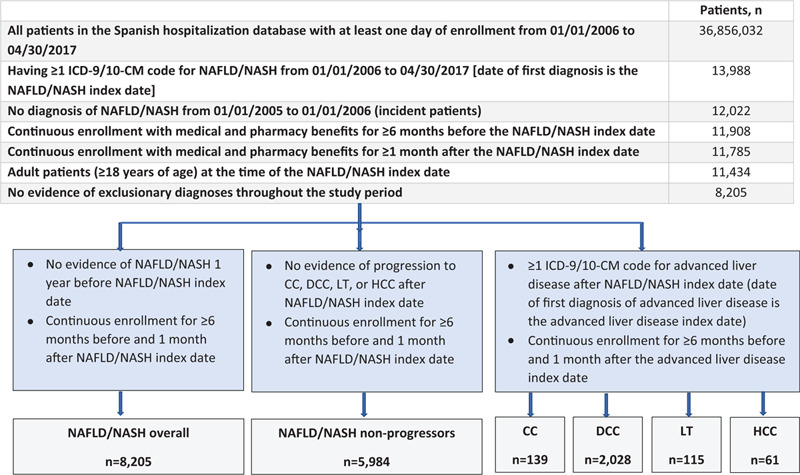
Patient disposition. CC, compensated cirrhosis; DCC, decompensated cirrhosis; HCC, hepatocellular carcinoma; LT, liver transplant; NAFLD, nonalcoholic fatty liver disease; NASH, nonalcoholic steatohepatitis.

The CMBD database included 36,856,032 patients for the period of 1 January 2006 through 30 April 2017, with 13,988 (0.04%) patients diagnosed with NAFLD/NASH (Fig. 1). After applying the inclusion and exclusion criteria, 8,205 patients were eligible for inclusion in this analysis. Of these, 5,984 (72.9%) did not progress to any advanced liver disease stage and were categorized as NAFLD/NASH non-progressors. A diagnosis of CC was confirmed in 139 (1.7%) patients, 2,028 (24.7%) were diagnosed with DCC, 115 (1.4%) had LT, and 61 (0.7%) progressed to HCC. Among patients diagnosed with cirrhosis (CC or DCC), 93.4% had a decompensated event at the time of their initial diagnosis of cirrhosis.

### 3.1 Demographic and clinical characteristics

The overall mean patient age was 58.4 years, ranging from 54.3 years for patients in the LT group to 70.9 years for those with HCC (Table 1). The majority of patients were male at 53.8% of all NAFLD/NASH patients, ranging from 52.2% of NAFLD/NASH non-progressors to 65.6% of those diagnosed with HCC.

**Table 1:** Baseline demographic and clinical characteristics.

	Severity of liver disease					
	NAFLD/NASH overall (n = 8,205)	NAFLD/NASH non-progressors (n = 5,984)	CC (n = 139)	DCC (n = 2,028)	LT (n = 115)	HCC (n = 61)
Demographics						
Age, yr						
Mean (SD)	58.4 (16.6)	54.8 (15.9)	65.2 (14.2)	68.8 (14.4)	54.3 (11.8)	70.9 (11.4)
Age group, yr, n (%)						
18–44	1,824 (22.2)	1,671 (27.9)	9 (6.5)	131 (6.5)	18 (11.6)	0 (0.0)
45–64	3,305 (40.3)	4,313 (72.1)	56 (40.3)	565 (27.9)	76 (49.0)	16 (26.2)
≥65	3,076 (37.5)	1,677 (28.0)	74 (53.2)	1,332 (65.7)	21 (13.6)	45 (73.8)
Sex, n (%)						
Female	3,793 (46.2)	2,859 (47.8)	57 (41.0)	878 (43.3)	24 (20.9)	21 (34.4)
Male	4,412 (53.8)	3,125 (52.2)	82 (59.0)	1,150 (56.7)	91 (79.1)	40 (65.6)
Region, n (%)						
Other	2,698 (32.9)	2,014 (33.7)	47 (33.8)	639 (31.5)	11 (9.6)	13 (21.3)
Andalusia	964 (11.7)	677 (11.3)	36 (25.9)	260 (12.8)	10 (6.5)	15 (24.6)
Catalonia	1,395 (17.0)	959 (16.0)	16 (11.5)	366 (18.1)	75 (48.4)	13 (21.3)
Galicia	687 (8.4)	561 (9.4)	5 (3.6)	123 (6.1)	0 (0.0)	2 (3.3)
Madrid	1,464 (17.8)	994 (16.6)	13 (9.4)	447 (22.0)	19 (12.3)	18 (29.5)
Valencian Community	997 (12.2)	779 (13.0)	22 (15.8)	193 (9.5)	0 (0.0)	0 (0.0)
Comorbid health conditions, n (%)						
Abdominal pain	178 (2.2)	158 (2.6)	0 (0.0)^∗^	19 (0.9)^∗^^,^^†^	1 (0.9)^∗^	0 (0.0)^∗^
Anemia	984 (12.0)	367 (6.1)	33 (23.7)^∗^^,^^†^	597 (29.4)^∗^^,^^†^	7 (6.1)^∗^^,^^†^	16 (26.2)^∗^^,^^†^
Apnea	123 (1.5)	92 (1.5)	1 (0.7)^∗^	31 (1.5)^∗^	0 (0.0)^∗^	1 (1.6)^∗^
Bariatric surgery	1 (0.0)	1 (0.02)	0 (0.0)	0 (0.0)	0 (0.0)	0 (0.0)
Cardiovascular disease	726 (8.8)	365 (6.1)	13 (9.4)^∗^	353 (17.4)^∗^^,^^†^	1 (0.9)^∗^^,^^†^	5 (8.2)^∗^^,^^†^
Diabetes mellitus, type 1	39 (0.5)	32 (0.5)	1 (0.7)^∗^	7 (0.4)^∗^	0 (0.0)^∗^	0 (0.0)^∗^
Diabetes mellitus, type 2	1,656 (20.2)	965 (16.1)	37 (26.6)^∗^	657 (32.4)^∗^^,^^†^	17 (14.8)^∗^^,^^†^	19 (31.1)^∗^^,^^†^
Dyspepsia	208 (2.5)	130 (2.2)	5 (3.6)^∗^	74 (3.7)^∗^^,^^†^	1 (0.9)^∗^	1 (1.6)^∗^
Hyperlipidemia	1,204 (14.7)	932 (15.6)	16 (11.5)^∗^	258 (12.7)^∗^^,^^†^	2 (1.7)^∗^^,^^†^	6 (9.8)^∗^^,^^†^
Hypertension	2,299 (28.0)	1,476 (24.7)	45 (32.4)^∗^	776 (38.3)^∗^^,^^†^	19 (16.5)^∗^^,^^†^	24 (39.3)^∗^^,^^†^
Insomnia	8 (0.1)	4 (0.07)	0 (0.0)	4 (0.2)	0 (0.0)	0 (0.0)
Obesity	569 (6.9)	424 (7.1)	12 (8.6)^∗^	136 (6.7)^∗^^,^^†^	5 (4.4)^∗^^,^^†^	0 (0.0)^∗^^,^^†^
Renal impairment	643 (7.8)	252 (4.2)	16 (11.5)^∗^	376 (18.5)^†^	11 (9.6)^∗^	10 (16.4)^∗^
Tobacco use, current or past	963 (11.7)	677 (11.3)	18 (12.9)^∗^	267 (13.2)^∗^^,^^†^	7 (6.1)^∗^^,^^†^	6 (9.8)^∗^^,^^†^
Thyroid disease	491 (6.0)	331 (5.5)	12 (8.6)^∗^	151 (7.5)^∗^^,^^†^	3 (2.6)^∗^^,^^†^	3 (4.9)^∗^^,^^†^
Vitamin D deficiency	32 (0.4)	26 (0.4)	0 (0.0)^∗^	6 (0.3)^∗^	0 (0.0)^∗^	0 (0.0)^∗^
Multiple comorbid health conditions, n (%)						
CVD AND diabetes (type 1 or 2) AND renal impairment	236 (2.9)	50 (0.8)	4 (2.9)^∗^	62 (3.1)^∗^^,^^†^	0 (0.0)^∗^	3 (4.9)^∗^
CVD OR diabetes (type 1 or 2) OR renal impairment	1,727 (21.0)	1,034 (17.3)	35 (25.2)^∗^	690 (34.1)^∗^^,^^†^	16 (13.9)^∗^^,^^†^	16 (26.2)^∗^^,^^†^
At least 1 of 5 comorbidities^¶^	3,648 (44.5)	1,190 (19.9)	33 (23.7)^∗^	738 (36.4)^∗^^,^^†^	17 (14.8)^∗^^,^^†^	18 (29.5)^∗^^,^^†^
At least 2 of 5 comorbidities^¶^	1,959 (23.9)	454 (7.6)	14 (10.1)^∗^	319 (15.7)^∗^^,^^†^	3 (2.6)^∗^^,^^†^	9 (14.8)^∗^
At least 3 of 5 comorbidities^‡^	782 (9.5)	108 (1.8)	4 (2.9)^∗^	101 (5.0)^∗^^,^^†^	0 (0.0)^∗^	3 (4.9)^∗^

CC = compensated cirrhosis, CVD = cardiovascular disease, DCC = decompensated cirrhosis, HCC = hepatocellular carcinoma, LT = liver transplant, NAFLD = nonalcoholic fatty liver disease, NASH = nonalcoholic steatohepatitis, SD = standard deviation.

∗ *P* < .05 for comparison with NAFLD/NASH non-progressors.

† *P* < .05 for comparison with CC.

‡ CVD, diabetes (type 1 or 2), hyperlipidemia, hypertension, renal impairment.

Baseline rates of comorbid health conditions were high overall and in each of the disease severity cohorts. The most common comorbidities were hypertension, type 2 diabetes, and hyperlipidemia. The prevalence of comorbid health conditions was higher in patients with advanced liver diseases compared with those in the NAFLD/NASH non-progressors group. Similarly, the frequency of multiple comorbidities increased significantly as patients progressed to advanced liver diseases (all *P* < .05).

### 3.2 Healthcare resource utilization

The mean number of admissions was numerically greater during the pre- and post-index periods for patients with CC, DCC, LT, and HCC compared to those in the NAFLD/NASH non-progressor group. There were no significant changes in the mean number of hospital readmissions during the post-index period compared with those during the pre-index interval within each of the severity cohorts.

**Figure 2:**
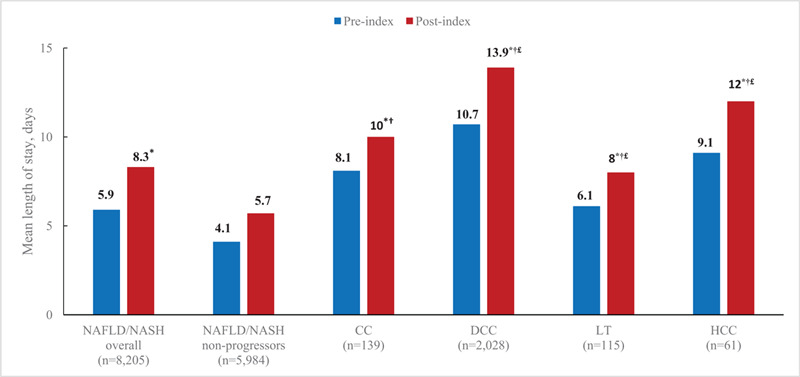
Mean length of stay per admission by severity of liver disease. ^∗^*P* < .05 for comparisons with post-index cost; ^†^*P* < .05 for comparison with post-index NAFLD/NASH non-progressors; ^£^*P* < .05 for comparison with post-index CC. CC, compensated cirrhosis; DCC, decompensated cirrhosis; HCC, hepatocellular carcinoma; LT, liver transplant; NAFLD, nonalcoholic fatty liver disease; NASH, nonalcoholic steatohepatitis.

The shortest mean LOS per admission at baseline was 4.13 days for NAFLD/NASH non-progressors. The pre-index LOS was significantly higher in each of the disease severity cohorts compared with NAFLD/NASH non-progressors at 8.11 days for CC patients, 10.74 days for DCC, days 6.12 for LT, and 9.08 days for HCC (Fig. 2). There was a numerical increase in mean LOS from the pre- to post-index period at 5.74 days for NAFLD/NASH non-progressors, although this was not statistically significant. Mean LOS during the post-index period was significantly higher than pre-index within each of the disease severity cohorts, increasing to 9.98 days for those with CC, 13.86 days for DCC, 8.01 days for LT, and 11.99 days for patients with HCC.

### 3.3 Healthcare costs

**Figure 3:**
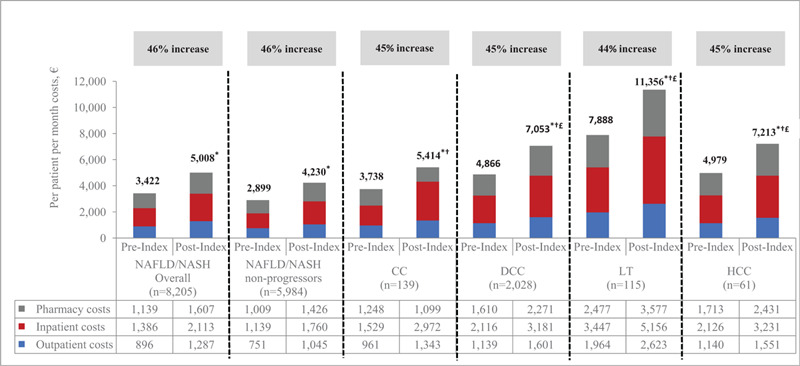
All-cause healthcare costs by severity of liver disease (unadjusted). ^∗^*P* < .05 for comparisons with post-index cost; ^†^*P* < .05 for comparison with post-index NAFLD/NASH non-progressors; ^£^*P* < .05 for comparison with post-index CC. CC, compensated cirrhosis; DCC, decompensated cirrhosis; HCC, hepatocellular carcinoma; LT, liver transplant; NAFLD, nonalcoholic fatty liver disease; NASH, nonalcoholic steatohepatitis.

Mean PPPM costs increased significantly from pre-index to post-index period (all *P* < .05) within each of the disease severity groups (Fig. 3). In addition, the post-index PPPM costs were significantly greater for:

(i) patients with advanced liver diseases as compared to NAFLD/NASH non-progressors (all *P* < .05) and
(ii) patients with DCC, LT, and HCC as compared to those with CC (all *P* < .05).

**Figure 4:**
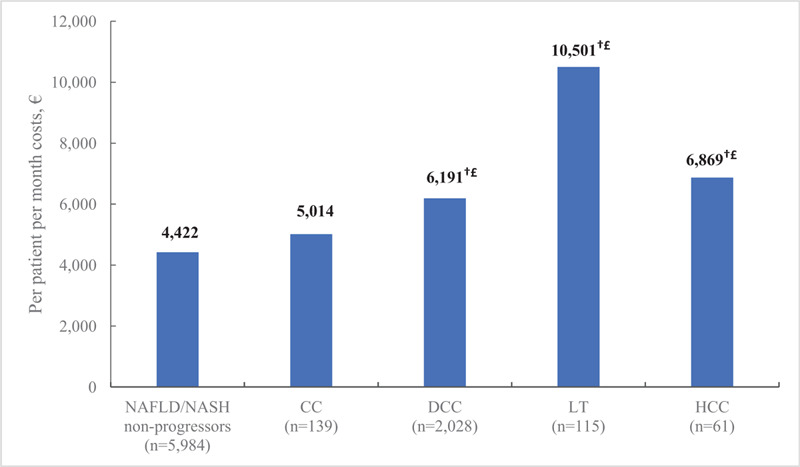
Post-index all-cause healthcare costs by severity of liver disease (adjusted∗). ^∗^Post-index adjusted costs were calculated via generalized linear model adjusted for age, sex, region, and comorbidities; ^†^*P* < .05 for comparison with post-index NAFLD/NASH non-progressors; ^£^*P* < .05 for comparison with post-index CC. CC, compensated cirrhosis; DCC, decompensated cirrhosis; HCC, hepatocellular carcinoma; LT, liver transplant; NAFLD, nonalcoholic fatty liver disease; NASH, nonalcoholic steatohepatitis.

Within each disease severity cohort, the cost increase from the pre-index to post-index periods was primarily associated with inpatient expenditures, which increased with the severity of liver disease. Inpatient PPPM costs during the post-index period accounted for 42% of the overall increase in healthcare expenditures for NAFLD/NASH non-progressors, 55% for those with CC, and 45% in the DCC, LT, and HCC cohorts. Significant predictors of higher, post-index costs were patient age and selected comorbid health conditions including renal impairment, sleep apnea, anemia, CVD, and tobacco use (Table 2). After adjusting for severity of liver disease, healthcare costs for patients with CC, DCC, LT, and HCC were significantly higher than those reported for the NAFLD/NASH non-progressor group (Fig. 4).

**Table 2:** Multivariable generalized linear model for adjusted post-index all-cause per patient per month healthcare costs.

Independent variable	Cost ratio	95% CI	*P*
Liver disease severity			
NAFLD/NASH non-progressor	Reference	–	–
CC	1.13	0.98, 1.32	.099
DCC	1.40	1.35, 1.45	.000^∗^
LT	2.37	2.10, 2.69	.000^∗^
HCC	1.55	1.32, 1.83	.000^∗^
Age group (yr)			
18–44	Reference	–	–
45–64	1.08	1.04, 1.12	.000^∗^
≥65	1.16	1.11, 1.21	.000^∗^
Sex			
Male	Reference	–	–
Female	1.01	0.98, 1.04	.427
Region			
Other	Reference	–	–
Andalusia	0.98	0.94, 1.03	.535
Catalonia	0.92	0.88, 0.96	.000^∗^
Galicia	0.96	0.90, 1.01	.107
Madrid	0.97	0.93, 1.02	.152
Valencian Community	0.89	0.85, 0.93	.000^∗^
Comorbid health condition			
Abdominal pain	0.93	0.84, 1.03	.145
Anemia	1.10	1.05, 1.15	.000^∗^
Apnea	1.12	0.99, 1.26	.062
Bariatric surgery	2.87	0.80, 10.36	.107
CVD	1.07	1.01, 1.13	.013^∗^
Diabetes mellitus, type 1	1.12	0.91, 1.38	.294
Diabetes mellitus, type 2	1.00	0.96, 1.04	.971
Dyspepsia	1.03	0.94, 1.13	.492
Hyperlipidemia	1.00	0.96, 1.05	.850
Hypertension	1.03	0.99, 1.07	.149
Insomnia	0.98	0.83, 1.13	.940
Obesity	1.05	0.99, 1.72	.131
Renal impairment	1.63	1.54, 1.72	.000^∗^
Tobacco use, current or past	1.05	1.00, 1.10	.033^∗^
Thyroid disease	1.00	0.94, 1.06	.978
Vitamin D deficiency	1.29	1.03, 1.62	.028^∗^

Reference: patients without comorbidity. CC = compensated cirrhosis, CI = confidence interval, CVD = cardiovascular disease, DCC = decompensated cirrhosis, HCC = hepatocellular carcinoma, LT = liver transplant, NAFLD = nonalcoholic fatty liver disease, NASH = nonalcoholic steatohepatitis.

∗ Statistical significance at *P* < .05.

## 4 Discussion

This real-world analysis of hospital admission data for patients with NAFLD/NASH confirms the substantial impact of NAFLD/NASH on healthcare systems in Spain. Hospital LOS for both the pre- and post-index periods was significantly longer for patients who progressed to advanced liver diseases compared to NAFLD/NASH non-progressors. The post-index PPPM costs within each of the disease severity groups were significantly higher for patients with advanced liver diseases than the non-progressors. The primary driver of cost increases from baseline to the post-index period were inpatient services, which accounted for 45% to 55% of the increase in expenditures for CC, DCC, LT, and HCC patients. Total costs for patients with advanced liver diseases remained higher compared to non-progressors after adjusting for demographic and clinical characteristics.

These findings are consistent with previous studies that report a substantial impact of NAFLD/NASH on healthcare resource utilization and costs, particularly for patients with advanced liver diseases.^[1,3,5,25–27,37,38,42–44]^ Two real-world analyses of administrative databases of Italian local health units and the French hospital system found that more severe liver disease was associated with higher mean total annual costs in hospitalized NAFLD/NASH patients. Inpatient services were the primary driver of higher costs, and costs were generally higher for patients with more advanced liver disease compared to those with less severe disease.^[38,43]^ The Global Assessment of the Impact of NASH (GAIN) study provided real-world estimates of the total annualized costs associated with NASH in France, Germany, Italy, Spain, the UK, and the US. The mean annual total direct costs for NASH patients in Spain were €3323.^[44]^ This was relatively lower than the costs reported in this study, which might be attributed to the inclusion of more ill, hospitalized patients as well as those with NAFLD in our study cohort.

A recent analysis of commercially insured patients in the US reported a 38.8% increase in the number of inpatient hospital admissions and a 61.8% increase in the mean number of annual admissions per patient following a diagnosis of CC. Per patient per year mean total healthcare costs rose from $25,720 (pre-diagnosis) to $35,715 (post-diagnosis).^[42]^ Similarly, a retrospective cohort analysis of NAFLD/NASH patients enrolled in US Medicare reported higher inpatient and outpatient charges for CC and DCC patients compared with noncirrhotic NAFLD patients. Those with DCC also had higher total inpatient and outpatient charges than CC patients.^[26]^

Many estimates of the economic impact of NAFLD/NASH in Europe and other countries have been generated by predictive models that are subject to limitations.^[1,3,5,13,27]^ Importantly, when compared to analyses based on real-world data, predictive models may underestimate the economic impact of NAFLD/NASH. A retrospective analysis of the MarketScan claims database reported adjusted annual all-cause healthcare costs that ranged from $23,860 for NAFLD/NASH with no progression to $197,392 for LT.^[42]^ Similarly, among NAFLD/NASH patients in Italy, annual healthcare costs, including pharmacy, outpatient, and inpatient services, ranged from €19,681 for CC patients to €65,137 for those undergoing LT.^[38]^ These costs are substantially higher than the annual predicted total direct medical cost per patient of $1,612 that was generated by models based on interlinked Markov chains.^[27]^ Other important limitations in the methodologies used to generate these predictive models include the use of cost estimates for managing other liver diseases as a proxy for costs in NAFLD/NASH patients; variability in the methods used to ascertain a clinical diagnosis of NAFLD and NASH; use of older data that may not accurately represent current patterns and trends in factors such as rates of obesity and diabetes; lack of consistency in techniques, terminology, and classification systems for fibrosis and cirrhosis stage; inclusion of data from studies that relied on small sample sizes or had short follow-up durations; and between-study variations in study aims and population characteristics.^[1,7]^

This real-world analysis provides a current and more accurate assessment of patterns of healthcare resource utilization and direct medical costs that are associated with NAFLD/NASH in Spain. Such information can be used to guide the development of interventions to improve the management of patients with NAFLD/NASH in the Spanish healthcare system as well as monitor the impact of such interventions.

This analysis highlighted the prevalence and impact of comorbidities in NALFD/NASH by severity of liver disease. Across the liver disease cohorts, we found that 15% to 45% of patients had at least 1 comorbid condition and the frequency of comorbid health conditions increased as patients progressed from NAFLD/NASH to advanced liver diseases. Previous studies have also reported an association between NAFLD/NASH and various comorbidities such as CVD,^[8,26]^ obesity, insulin resistance, type 2 diabetes, hypertension, and hyperlipidemia, with each of these disorders characteristic of the metabolic syndrome.^[6,8–11,14,16,17,38,45,46]^ In this Spanish population, we found that renal impairment, anemia, sleep apnea, and smoking were also associated with increased severity of liver disease and may have contributed to the higher costs and service utilization observed in these patients. Ongoing monitoring and management of all comorbid health conditions, including those that might be more typical of patients receiving care in Spain, may allow public health approaches to be targeted to the specific risk profile of patients in the Spanish healthcare system and may optimize the use of healthcare resources and funding.

As the prevalence of NAFLD/NASH and associated comorbidities continues to increase worldwide, the need for public health interventions and initiatives that are tailored to the unique demographic and clinical characteristics of patients residing in diverse regions of the world will be of critical importance.^[6,7,47]^ Such an approach will rely on improved identification of patients with progressive disease in the absence of symptoms, initiation of pharmacological and lifestyle interventions to address the metabolic and cardiovascular comorbidities that are characteristic of NAFLD/NASH, and promotion of recommended lifestyle interventions to prevent or slow disease progression. Optimal clinical management combined with efforts to reduce and manage risk factors for mortality and disease progression have the potential to decrease healthcare resource utilization and costs, especially when considered over the duration of disease. Such efforts are particularly important in the absence of noninvasive screening and diagnostic tests for NAFLD and approved pharmacotherapies for the treatment of NAFLD/NASH.

Consideration of regional and between-country variations in demographics, geography, and historical factors will be essential when planning a comprehensive program for Spain to address the current and future impact of NAFLD/NASH. A cross-sectional study reported an overall prevalence for NAFLD of 25.8% in adult patients receiving treatment at primary care centers in Catalonia, Spain, ranging from 20.3% in women to 33.4% in men.^[48]^ Important limitations of this study were a 60% nonparticipation rate and reliance on echography for the diagnosis of NAFLD. The current study provides a more accurate understanding of the epidemiologic burden of NAFLD/NASH, including factors that might be associated with poor clinical outcomes and a greater need for healthcare resources. This information can form the basis of a strong NAFLD/NASH public health initiative in Spain, with the potential to reduce the future burden of NAFLD/NASH.

The retrospective study design and use of a hospital discharge records database, which relies on administrative claims data for disease identification and assessment, are 2 important limitations of this analysis. Data for the CMBD are primarily collected for accounting purposes, and the information is subject to data coding limitations, data entry errors, and misclassification of NAFLD/NASH and liver disease severity. These factors could explain, in part, the low rate of CC in this analysis. In addition, the CMBD may not be fully representative of the entire population of Spain, and the patterns of healthcare resource utilization and costs in this study might not apply to the general population of Spanish patients with NAFLD/NASH who are not included in the CMBD.

The identification of patients with advanced liver diseases was limited to ICD-CM-09 and ICD-CM-10 codes rather than laboratory or biopsy data or other measures of fibrosis such as elastography and ultrasound. This may have resulted in an underestimation of the true number of patients who experienced disease progression in the case of asymptomatic disease progression. Patients might have had advanced liver disease before their initial NASH/NAFLD diagnosis, and the non-progressor group might have included F0 to F3 patients as well as undiagnosed F4 (CC) patients due to under-coding and lack of ICD codes for F0 to F3. Moreover, since CMBD includes hospitalized patients in Spain, the estimates for severe liver diseases could be inflated due to patients being comparatively sicker. Readmission was defined as an admission to the same hospital within 30 days of discharge, and patients who were admitted to other hospitals during that time period might have not have been identified, which would underestimate hospital readmission rates. Several potentially important factors were controlled for in these multivariable analyses, but adjustment was limited to characteristics that could be measured with data that were available.

While these are important limitations, this study has several strengths. The analysis was based on a large cohort of NAFLD/NASH patients at varying stages of disease severity, and patients’ non-progression or progression through CC, DCC, LT, and HCC was tracked. The inclusion criteria required patients to have at least 1 month follow-up after the post-index date. This criterion preserved sample size and might have reduced bias associated with a longer follow-up interval in healthier patients who did not die or progress to a more advanced stage of liver disease.

In conclusion, patients with advanced liver diseases were more likely to experience one or more comorbid health conditions, use more healthcare resources, and incur higher costs compared to those with no evidence of disease progression. The primary cost drivers were associated with inpatient services. These findings highlight opportunities to improve overall patient management and ensure optimal allocation of healthcare resources and control costs associated with NAFLD/NASH in Spain.

## Acknowledgments

Writing assistance was provided by Carole Alison Chrvala, PhD, Health Matters, Inc. and financially supported by Gilead Sciences Inc.

## Author contributions

**Conceptualization:** Manuel Romero-Gomez, Nandita Kachru, Meritxell Ascanio Zamorano, Josep Darba, Sanatan Shreay.

**Data curation:** Manuel Romero-Gomez, Nandita Kachru, Meritxell Ascanio Zamorano, Josep Darba, Sanatan Shreay.

**Formal analysis:** Manuel Romero-Gomez, Nandita Kachru, Meritxell Ascanio Zamorano, Josep Darba, Sanatan Shreay.

**Investigation:** Manuel Romero-Gomez, Nandita Kachru, Meritxell Ascanio Zamorano, Josep Darba, Sanatan Shreay.

**Methodology:** Manuel Romero-Gomez, Nandita Kachru, Meritxell Ascanio Zamorano, Josep Darba, Sanatan Shreay.

**Project administration:** Manuel Romero-Gomez, Nandita Kachru, Meritxell Ascanio Zamorano, Josep Darba, Sanatan Shreay.

**Resources:** Manuel Romero-Gomez, Nandita Kachru, Meritxell Ascanio Zamorano, Josep Darba, Sanatan Shreay.

**Supervision:** Manuel Romero-Gomez, Nandita Kachru, Meritxell Ascanio Zamorano, Josep Darba, Sanatan Shreay.

**Validation:** Manuel Romero-Gomez, Nandita Kachru, Meritxell Ascanio Zamorano, Josep Darba, Sanatan Shreay.

**Visualization:** Manuel Romero-Gomez, Nandita Kachru, Meritxell Ascanio Zamorano, Josep Darba, Sanatan Shreay.

**Writing – original draft:** Manuel Romero-Gomez, Nandita Kachru, Meritxell Ascanio Zamorano, Josep Darba, Sanatan Shreay.

**Writing – review & editing:** Manuel Romero-Gomez, Nandita Kachru, Meritxell Ascanio Zamorano, Josep Darba, Sanatan Shreay.

## Supplementary Material

Supplemental Digital Content
