# Supplementary material for: Disease severity predicts higher healthcare costs among hospitalized nonalcoholic fatty liver disease/nonalcoholic steatohepatitis (NAFLD/NASH) patients in Spain
Source: Medicine (Baltimore). 2020 Dec 11;99(50):e23506. doi: 10.1097/MD.0000000000023506 (PMC7738099; doi:10.1097/MD.0000000000023506)
Supplement: Supplemental Digital Content [file medi-99-e23506-s001.docx]

Appendix 1. List of International Classification of Disease, Ninth Revision, Tenth Revision, Clinical Modification Codes

ICD-9-CM and ICD-10-CM codes for nonalcoholic fatty liver disease and nonalcoholic steatohepatitis

| Code | Code Type | Description |
| --- | --- | --- |
| 571.8 | ICD-9 Dx | Other chronic nonalcoholic liver disease |
| 571,9 | ICD-9 Dx | Unspecified chronic liver disease without mention of alcohol |
| K760 | ICD-10 Dx | Fatty (change of) liver, not elsewhere classified |
| K7581 | ICD-10 Dx | Nonalcoholic steatohepatitis (NASH) |

ICD-9-CM and ICD-10-CM codes for compensated cirrhosis

| Code | Code Type | Description |
| --- | --- | --- |
| 571.5 | ICD-9 Dx | Cirrhosis of liver without mention of alcohol |
| 456.1 | ICD-9 Dx | Esophageal varices without mention of bleeding |
| K74.60 | ICD-10 Dx | Unspecified cirrhosis of liver |
| K74.69 | ICD-10 Dx | Other cirrhosis of liver |
| I85.00 | ICD-10 Dx | Esophageal varices without bleeding |
| I85.10 | ICD-10 Dx | Secondary esophageal varices without bleeding |

ICD-9-CM and ICD-10-CM codes for decompensated cirrhosis

| Code | Code Type | Description |
| --- | --- | --- |
| 348.3 | ICD-9 Dx | Encephalopathy. not elsewhere specified |
| 348.31 | ICD-9 Dx | Metabolic encephalopathy |
| 348.39 | ICD-9 Dx | Other encephalopathy |
| 456.0 | ICD-9 Dx | Esophageal varices with bleeding |
| 456.2 | ICD-9 Dx | Esophageal varices with bleeding in diseases classified elsewhere |
| 483.3 | ICD-9 Dx | Encephalopathy. not elsewhere specified |
| 511.89 | ICD-9 Dx | Other specified forms of effusion. except tuberculosis (hepatic hydrothorax) |
| 511.9 | ICD-9 Dx | Unspecified pleural effusion (hepatic hydrothorax) |
| 530.82 | ICD-9 Dx | Esophageal hemorrhage |
| 567.23 | ICD-9 Dx | Spontaneous bacterial peritonitis |
| 568.82 | ICD-9 Dx | Peritoneal effusion (chronic) |
| 572.2 | ICD-9 Dx | Hepatic encephalopathy |
| 572.4 | ICD-9 Dx | Hepatorenal syndrome |
| 573.5 | ICD-9 Dx | Hepatopulmonary syndrome |
| 578.0 | ICD-9 Dx | Hematemesis |
| 578.1 | ICD-9 Dx | Blood in stool |
| 578.9 | ICD-9 Dx | Hemorrhage of gastrointestinal tract |
| 782.4 | ICD-9 Dx | Jaundice. unspecified. not of newborn |
| 789.5 | ICD-9 Dx | Ascites |
| 789.59 | ICD-9 Dx | Ascites (other) |
| K7201 | ICD-10 Dx | Acute and subacute hepatic failure with coma |
| K7200 | ICD-10 Dx | Acute and subacute hepatic failure without coma |
| K762 | ICD-10 Dx | Central hemorrhagic necrosis of liver |
| K7211 | ICD-10 Dx | Chronic hepatic failure with coma |
| K7210 | ICD-10 Dx | Chronic hepatic failure without coma |
| G9340 | ICD-10 Dx | Encephalopathy, not elsewhere specified |
| K228 | ICD-10 Dx | Esophageal hemorrhage |
| I8501 | ICD-10 Dx | Esophageal varices with bleeding |
| K922 | ICD-10 Dx | Gastrointestinal hemorrhage, unspecified |
| K920 | ICD-10 Dx | Hematemesis |
| K7291 | ICD-10 Dx | Hepatic failure, unspecified with coma |
| K7290 | ICD-10 Dx | Hepatic failure, unspecified without coma |
| K7681 | ICD-10 Dx | Hepatopulmonary syndrome |
| K767 | ICD-10 Dx | Hepatorenal syndrome |
| R17 | ICD-10 Dx | Jaundice, unspecified, not of newborn |
| K921 | ICD-10 Dx | Melena |
| G9341 | ICD-10 Dx | Metabolic encephalopathy |
| R188 | ICD-10 Dx | Other ascites |
| G9349 | ICD-10 Dx | Other encephalopathy |
| J90 | ICD-10 Dx | Pleural effusion, nec (hepatic hydrothorax) |
| I8511 | ICD-10 Dx | Secondary esophageal varices with bleeding |
| K652 | ICD-10 Dx | Spontaneous bacterial peritonitis |

ICD-9-CM and ICD-10-CM codes for hepatocellular carcinoma

| Code | Code Type | Description |
| --- | --- | --- |
| 155.0 | ICD-9 Dx | Malignant neoplasm of liver, primary |
| 155.1 | ICD-9 Dx | Malignant neoplasm of intrahepatic bile ducts |
| C22.0 | ICD-10 Dx | Liver cell carcinoma |
| C22.1 | ICD-10 Dx | Intrahepatic bile duct carcinoma |
| C22.8 | ICD-10 Dx | Malignant neoplasm of liver, primary, unspecified as to type |
| C24.0 | ICD-10 Dx | Malignant neoplasm of extrahepatic bile duct |

ICD-9-CM and ICD-10-CM codes for liver transplant

| Code | Code Type | Description |
| --- | --- | --- |
| 50.51 | ICD-9 Proc | Auxiliary liver transplant |
| 50.59 | ICD-9 Proc | Other transplant of liver |
| 996.82 | ICD-9 Dx | Complications of transplanted liver |
| V42.7 | ICD-9 Dx | Liver replaced by transplant |
| 0FY00Z0 | ICD-10 Proc | Transplantation of Liver, Allogeneic, Open Approach |
| 0FY00Z1 | ICD-10 Proc | Transplantation of Liver, Syngeneic, Open Approach |
| 0FY00Z2 | ICD-10 Proc | Transplantation of Liver, Zooplastic, Open Approach |
| T86.40 | ICD-10 Dx | Unspecified complication of liver transplant |
| T86.41 | ICD-10 Dx | Liver transplant rejection |
| T86.42 | ICD-10 Dx | Liver transplant failure |
| T86.43 | ICD-10 Dx | Liver transplant infection |
| T86.49 | ICD-10 Dx | Other complications of liver transplant |
| Z48.23 | ICD-10 Dx | Encounter for aftercare following liver transplant |
| Z94.4 | ICD-10 Dx | Liver transplant status |
